# Vascular Occlusion after Hyaluronic Acid Filler Injection

**DOI:** 10.5811/cpcem.2018.2.37149

**Published:** 2018-04-05

**Authors:** Sarah Grzybinski, Elizabeth Temin

**Affiliations:** Massachusetts General Hospital, Department of Emergency Medicine, Boston, Massachusetts

## CASE PRESENTATION

A 26-year-old female presented to the emergency department (ED) with progressively worsening forehead erythema and discomfort after receiving an injection of hyaluronic acid filler into the glabella at a medical spa the previous day. On arrival to the ED she was afebrile with normal vital signs. Physical examination revealed blanchable erythema extending from the mid-forehead to the nasal bridge in a linear distribution with early retiform appearance on the nasal sidewalls (Image). The patient denied visual symptoms, and ophthalmic examination was normal. She was diagnosed with vascular occlusion as a result of hyaluronic acid filler injection.

She was treated with an intradermal injection of 490U hyaluronidase (2.45mL of 200U/mL) administered in multiple small injections around the linear erythema. After the injection, a single dose of 2% nitroglycerin paste was applied to the skin for five minutes. She was discharged home on a two-week course of aspirin 325mg daily. The patient had outpatient follow-up one day and four days after her initial presentation. She had complete resolution of symptoms.

## DISCUSSION

Vascular occlusion is a rare but potentially detrimental complication of hyaluronic acid filler injections. It results from accidental injection of filler into an artery, or compression of the artery from surrounding filler.^1^ Vascular occlusion can rapidly progress to tissue necrosis if not identified and treated quickly. Retinal branch artery occlusion is also a potential complication of filler injections, caused by retrograde arteriolar flow of the filler into the branches of the ophthalmic artery.^2^ It is imperative to ask about visual symptoms and perform a thorough ophthalmic examination in these patients. The treatment of hyaluronic acid-induced vascular occlusion involves intradermal injection of hyaluronidase, a protein enzyme that degrades hyaluronic acid. Patients may also benefit from topical nitroglycerin paste to facilitate vasodilation, although this is controversial. Experts also recommend a short-term aspirin regimen to prevent further clotting and vascular compromise.^3^

**CPC-EM Capsule**

What do we already know about this clinical entity?

Vascular occlusion is a rare but serious complication of intradermal filler injections that can rapidly lead to tissue necrosis if not identified and treated quickly.

What is the major impact of the image(s)?

As intradermal filler injections are becoming more readily available, it is important to recognize the clinical features of vascular occlusion.

How might this improve emergency medicine practice?

Keep vascular occlusion high on the differential in patients presenting after filler injection. Consider treatment with intradermal hyaluronidase, topical nitroglycerin, and oral aspirin.

Documented patient informed consent and/or Institutional Review Board approval has been obtained and filed for publication of this case report.

## Figures and Tables

**Image:**
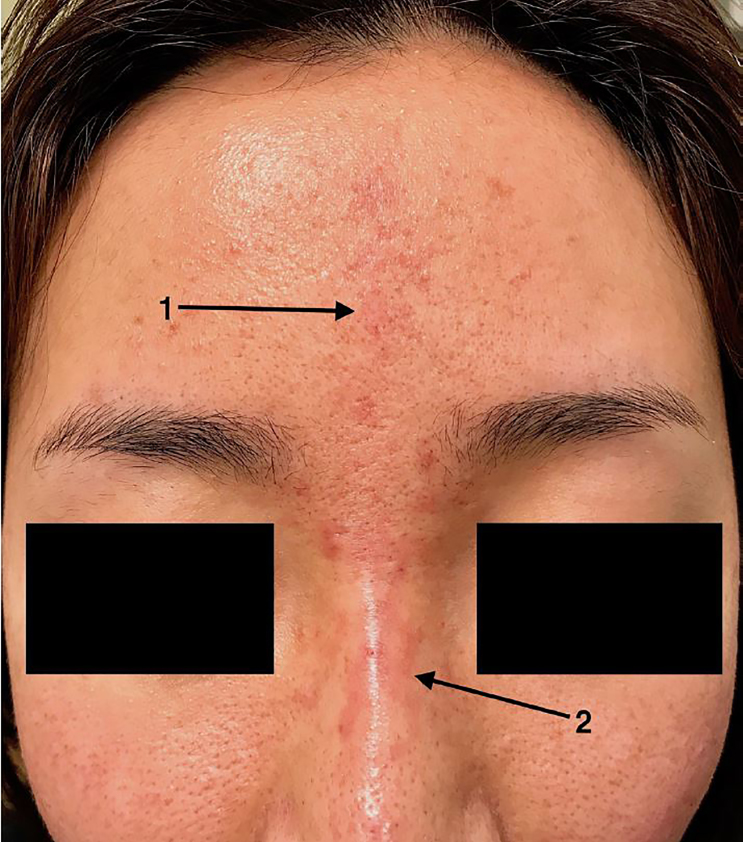
Linear erythema of the forehead (arrow 1) with retiform appearance of the nasal bridge (arrow 2) in a woman after hyaluronic acid filler injection.
